# Temperature-Phase Converter Based on a LC Cell as a Variable Capacitance

**DOI:** 10.3390/s150305594

**Published:** 2015-03-06

**Authors:** Juan Carlos Torres, Braulio García-Cámara, Isabel Pérez, Virginia Urruchi, José Manuel Sánchez-Pena

**Affiliations:** Displays and Photonic Applications Group, Department of Electronic Technology, Carlos III University of Madrid, Avenida de la Universidad 30, Leganés E28911, Spain; E-Mails: brgarcia@ing.uc3m.es (B.G.-C.); isaper@ing.uc3m (I.P.); vurruchi@ing.uc3m.es (V.U.); jmpena@ing.uc3m.es (J.M.S.-P.)

**Keywords:** nematic liquid crystal, impedance analysis, temperature dependence, equivalent circuit, capacitive sensor, simulation

## Abstract

The main characteristic of liquid crystals is that their properties, both electrical and optical, can be modified through a convenient applied signal, for instance a certain voltage. This tunable behavior of liquid crystals is directly related to the orientation of their nanometric components with respect to a director direction. However, the initial alignment is a fabrication-dependent parameter and may be either planar or homeotropic. In addition, the strong dependence of the properties of liquid crystals with the temperature is well known and widely used for several temperature sensors. This dependence is produced by the influence of the temperature on the ordering of the molecules. In this work, we have studied the temperature dependence of the electric properties of a liquid crystal cell, in particular the dielectric permittivity, with the temperature as a function of the initial alignment set during the fabrication process. Starting from experimental measurements, an equivalent circuit model including the temperature dependence has been proposed. We have observed that a good linearity in a wide temperature range is provided at a suitable exciting frequency. Finally, a proper conditioner circuit is proposed as a powerful tool for linear and high sensibility temperature measurement.

## 1. Introduction

Liquid crystals (LCs) are composed of nanometric-sized elongated organic molecules. This particular shape allows the molecules to exhibit orientational order in a particular direction under appropriate conditions [1]. A consequence of this ordering is an intrinsic anisotropy of their mechanical, electrical, magnetic and optical properties [2]. Their electrical and optical anisotropy, which are easily modified by applying an external stimulus, such as an electric field, make them unique for display applications [3]. In this sense, liquid crystals displays or LCDs are widespread, to the extent that we can found LCDs in applications ranging from mobile phones to digital cinema. Furthermore, other very interesting optical applications based on liquid crystals have emerged recently which have motivated a deeper knowledge and technological capabilities to manipulate them. We can highlight, for instance, wavelength tunable filters [4], variable optical attenuators [3], spatial light modulators [5], optical multiplexers [6], or optical adaptative systems [7], among others. LCs are also present in new research lines in photonics such as plasmonics [8,9] and metamaterials [10]. In addition, the tunable properties of LC can also be used for non-optical applications. For instance, we showed in previous works the use of LC cells to implement electronic devices such as sinusoidal oscillators [11], phase-locked loops (PLLs) [12] or tunable series-parallel resonators [13].

Within all these applications, the use of LC cells as a temperature sensor is also quite interesting. The strong influence of temperature in common processes of several fields, from industry to biology, requires accurate measurements of this parameter. Currently, there is a wide range of temperature sensors, from thermistors [14] to current temperature fiber sensors [15]. The advantage of LCs is the high sensitivity of their properties to any temperature change, through a high thermo-optic coefficient [16,17]. However, in a commercial device, including a LC cell, the fabrication conditions, in particular the initial alignment of the LC, are significant in the temperature sensitivity of the device.

In this work, the sensitivity of the electrical properties of nematic liquid crystal (NLC) cells has been studied as a function of alignment, either homeotropic or planar, and the thickness of the cell. In addition, an electrical equivalent circuit is proposed to analyze the temperature dependence through their parameters. As it will be shown, the detailed analysis of this model allows the optimization of important parameters, such as linearity and sensitivity, for the design of LC-based temperature sensors.

## 2. Experimental Section

**Figure 1:**
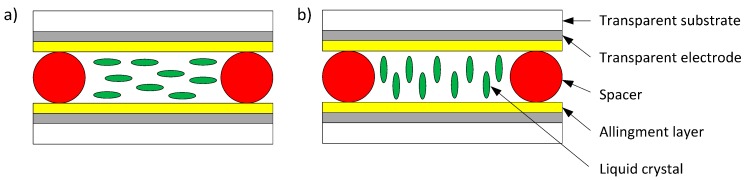
Schematic illustration of alignment in nematic liquid crystal cells, V_RMS_ = 0 V: (**a**) planar alignment; (**b**) homeotropic alignment.

Experimental samples were fabricated using the liquid crystal known as MLC-6290, which has a positive dielectric anisotropy (Δε > 0). This is a commercial material (Merck, Darmstadt, Germany) with an optical birefringence Δn = 0.12@588nm, a viscosity of 20 mm^2^·s^−1^ (T = 20 °C) and a clearing point of 104 °C. NLC is sandwiched in a cell composed of two glass plates with an area of 1 cm^2^ and covered with a conductive film. In particular, we have considered a layer of indium-tin-oxide (ITO) as conductive medium. The gap between both plates was fixed using spacers located along the perimeter of the plate area. Spacer balls were used to ensure that the glass plates remain a certain distance spaced over the entire area of the device. The cells were fabricated with different thickness, 6.3 μm and 1.5 μm, approximately. In addition, samples were prepared with two different alignments. For a planar alignment LC molecules were oriented parallel to the surface of the layer (Figure 1a) using rubbed polyimides in the alignment layer [3]. In this case, the presence of an external electric field can induce a reorientation of the molecules parallel to this field. On the other hand, when we consider a homeotropic alignment, LC molecules are perpendicular to the surface of the layer (Figure 1b), due to the presence of a monolayer surfactants (silane) and the molecules cannot be reoriented due to the presence of an external field. Spacer balls may affect the local alignment of LC molecules. However, both optical (polarization) and electrical (impedance) measurements were carried out to ensure a correct alignment, from a macroscopic point of view.

The values of the components of the considered electrical equivalent circuit that we propose are obtained through complex impedance measurements. The complex impedance (magnitude and phase) of LC devices has been measured with an impedance analyzer (SOLARTRON 1260, West Sussex, UK) using a sinusoidal voltage signal with 100 mV_RMS_ and frequency sweeping in a frequency range of 100 Hz to 10 MHz. The value of the applied voltage was chosen after several tests in order to avoid any influence in the LC and to achieve a high resolution in the measurements. In addition, in order to analyze properly the influence of the temperature on these parameters, samples were placed in a programmable environmental chamber (CCK-40/180, DYCOMETAL, Barcelona, Spain) to ensure a stable ambient temperature. Further, this temperature can be controlled in a range from 0 to 80 °C in steps of 5 °C. This maximum temperature was chosen in order to prevent the proximity of the clearing point, at which the LC changes its phase. Measurements were carried out in a steady state, with a smooth variation of the temperature (0.4 °C/min) in order to avoid any thermal expansion effects [18].

## 3. Results and Discussion

### 3.1. Characterization of the LC Cells

**Figure 2:**
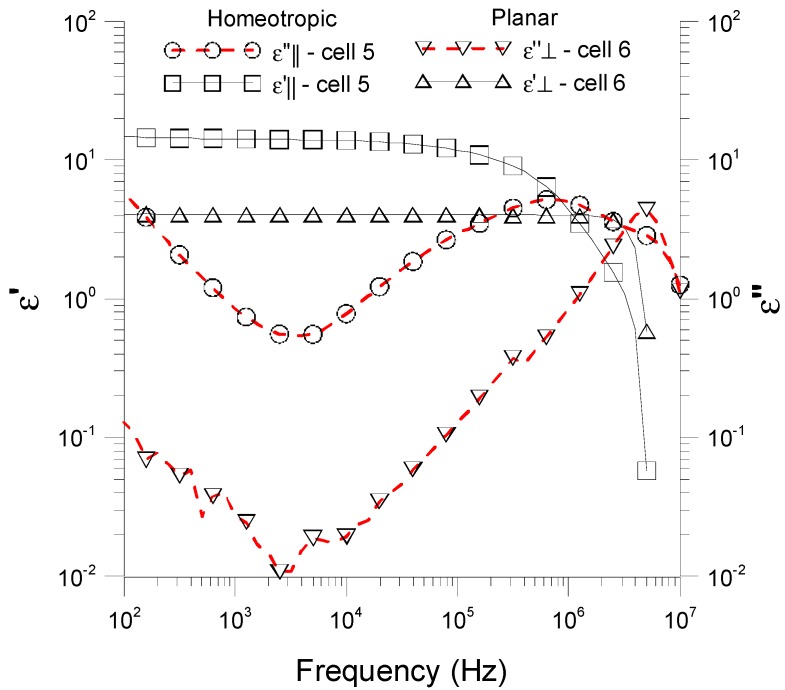
Experimental data of the complex dielectric permittivity of NLC 6290 cells as a function of the frequency of an applied electric field and for both planar and homeotropic cells, at a room temperature of 30 °C.

As it is well known, nematic liquid crystals are dielectric materials and their dielectric permittivity (*ɛ = ɛ*' *+ jɛ*'') can be written as a complex quantity, where *ɛ*' is the real part and *ɛ*'' is the dielectric loss factor [19,20]. As aforementioned, these values are strongly dependent on the orientation of the molecules of the LC. For this reason, first of all, we characterized our experimental samples measuring the electric permittivity, both the real and the imaginary part, as a function of the frequency of an applied electric field and for both kind of the considered alignments (Figure 2) at room temperature (~30 °C). These experimental data were obtained through impedance measurements. Although we built a large number of samples, all of them with similar response, we only show results for some of them, due to space constraints and to ensure clarity. In this case, we have considered cells called *cell 5* and *cell 6*. While *cell 5* has a homeotropic alignment, the molecules of *cell 6* are oriented parallel to the substrate (planar alignment). In both cases, the thickness of the cell is 6.3 μm.

As can be shown, a similar behavior could be observed for both planar and homeotropic cells. However, due to the positive dielectric anisotropy of MLC-6290, the values of the *parallel* (homeotropic) dielectric permittivity are greater than the *perpendicular* (planar) dielectric permittivity. Low frequencies (*f <* 100 Hz) are discarded from these measurements because the electric field can induce degradation of the LC material due to the absorption of ion charges and generation of electric field on the electrode layers [21,22]. On the other hand, at high frequencies, the imaginary part of the dielectric permittivity (*ɛ*'') presents a maximum. This corresponds to an absorption peak due to the dipolar relaxation. While it appears at 8 MHz for the planar cell (*cell 6*), for the homeotropic one (*cell 5*) the maximum is located almost a decade below (~800 kHz). A feasible explanation of this fact is that less energy is required to produce molecular motion in planar alignment than in homeotropic alignment [23]. In addition, within the medium range, the curves present two regions, with a different dielectric behavior, separated by the minimum reached by the imaginary part of the dielectric permittivity at 3 kHz, for both samples [24]. These minima will be analyzed from an impedance point of view below.

### 3.2. Equivalent Circuit Model

**Figure 3:**
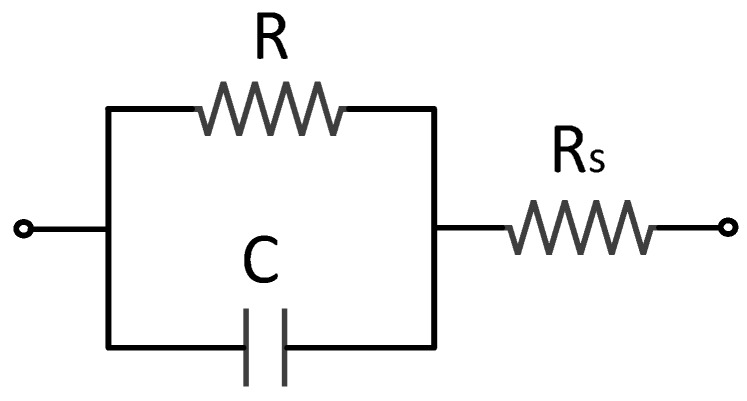
Equivalent electric circuit of a LC cell for a frequency range of 100 to 10^5^ Hz.

Based on the previous results, we consider that our samples are electrically equivalent to a circuit like that plotted in Figure 3 at medium frequencies (100 Hz *< f <* 10^5^ Hz). This model includes a capacitor C representing the dipolar polarization and the symmetry of the device and a resistor R modeling the mobility of free charges and the dipolar displacement inside the device. In addition, the influence of electrodes is represented with an extra resistor, *Rs*. However, the effect of this component is only appreciable at higher frequencies (*f >* 3 MHz).

The value of each component of this equivalent circuit as a function of the frequency could be inferred from the complex dielectric permittivity of Figure 2 by means of Equations (1) and (2):

(1) $$R(\omega )=\frac{1}{\omega \cdot \varepsilon^{\prime \prime }\cdot C_{0}}$$

(2) $$C=\varepsilon^{\prime }\cdot C_{0}$$

C_0_ being the vacuum device capability.

### 3.3. Temperature Dependence of the Equivalent Electric Circuit

As was mentioned above, the dielectric permittivity of NLCs depends considerably on temperature. In positive NLCs (Δε *>* 0), such as MLC-6290, this dependence is stronger for the parallel component (ε_||_) than for the perpendicular one (ε_⊥_) [22]. This effect could also be modeled through the values of the components of the equivalent circuit. The temperature dependence is noticed in both the resistor and the capacitor due to the variation of both the mobility and density of the ions as the temperature changes. However we have focused our attention on the capacitance part. The main reason to limit the study is that at a dominant capacitance behavior, the power consumption is also minimum, which is an important characteristic for potential future devices. In addition, this assumption is translated into negligible values of the conductivity.

**Figure 4:**
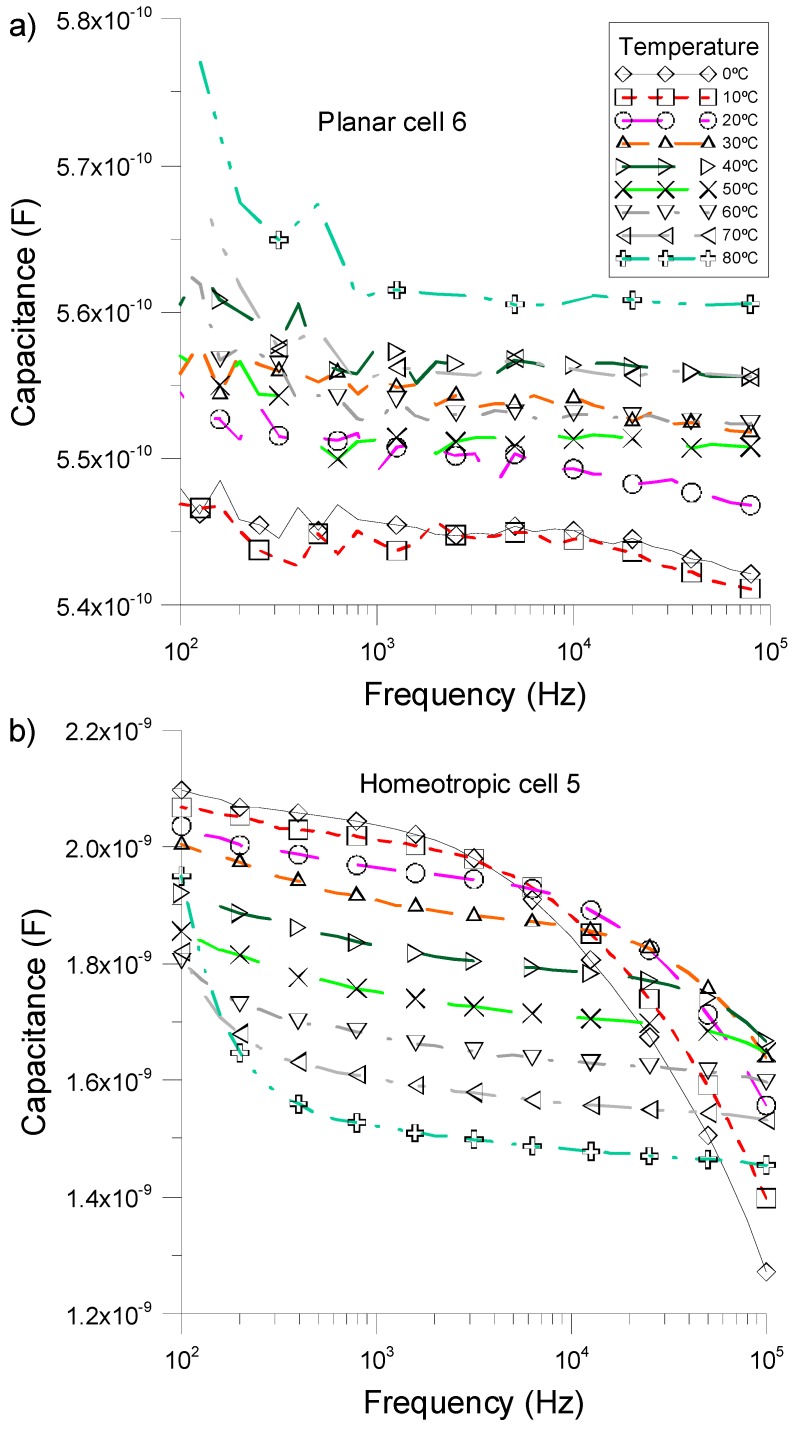
Variation of capacitance (C) as a function of the external frequency for different temperature values to (**a**) NLC *cell 6* (planar cell) and (**b**) NLC *cell 5* (homeotropic cell).

By measuring the dielectric permittivity of the samples for several temperatures, we calculated the value of the equivalent capacitor. Figure 4 shows the corresponding values of samples *cell 5* and *cell 6* as a function of the external frequency and for temperatures ranging from 0 to 80 °C. As it was expected, the capacitance in both cases decreases with temperature, because the mobility and the numerical density of the ions increase with temperature. However, this change is quite different for both samples. While in the planar cell (Figure 4a) this variation is approximately 0.25 pF/°C, at *f* = 1 kHz, in the homeotropic one (Figure 4b) it is around 35 times higher (~8.75 pF/°C). Thus, we can conclude that devices with a homeotropic alignment are more sensitive to temperature changes. In addition, in absence of an applied external voltage, while planar samples are not sensitive to temperature variations due to horizontal alignment, homeotropic samples are vertically aligned without any external field, producing a considerable sensitiveness.

**Figure 5:**
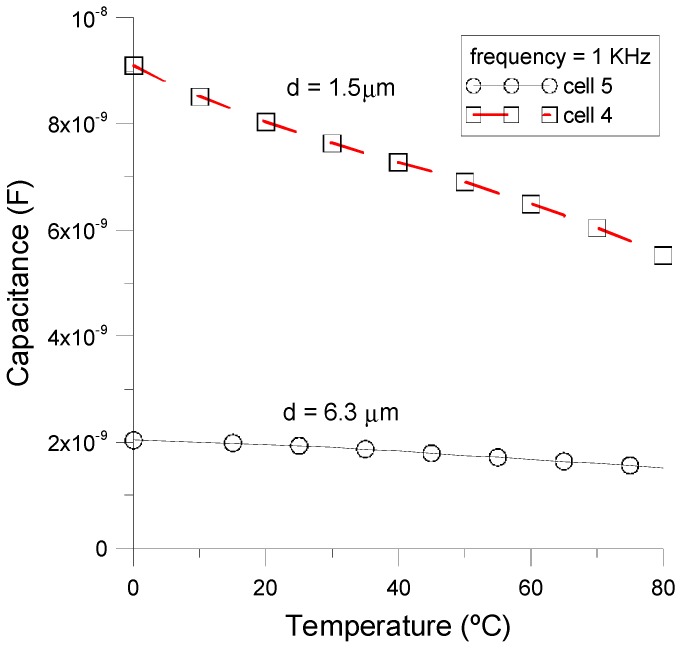
Temperature dependence of the liquid crystal capacitance taken in homeotropic cells with different thickness: *cell 5* (thickness = 6.3 μm) and *cell 4* (thickness = 1.5 μm).

In order to perform a detailed analysis of this behavior, the dependence of equivalent capacitance with the temperature could be analytically expressed as:

(3) $$S_{C}=\frac{\varepsilon_{0}\cdot S}{d}\cdot \frac{\partial \varepsilon \prime }{\partial T}$$

where *S_C_* is given in units of pF/°C. ɛ_0_ is the vacuum permittivity, *S* is the effective area of the electrodes, and *d* is the thickness of the NLC cell. As can be seen, by decreasing the thickness of the device, the sensitivity should increase linearly. Figure 5 shows the sensitivity (*S_C_*) of two homeotropic cells with different thicknesses, 1.5 μm (*cell 4*) and 6.3 μm (*cell 5*), and considering a frequency of 1 kHz. It can be noted that the relative variation of capacitance in *cell 4*, which is thinner than *cell 5*, is larger than in *cell 5* demonstrating the relation of Equation (3).

**Figure 6:**
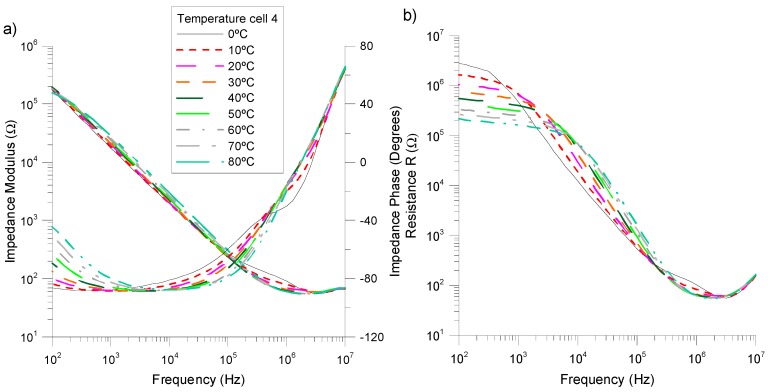
(**a**) Experimental complex impedance and (**b**) resistance of the NLC *cell 4* as function of frequency for a temperature range of 0 °C to 80 °C.

Previously, we considered a dominance of the capacitive character in the equivalent circuit model in order to obtain a simplified response. However, this is not suitable for any frequency. The frequency range at which the system acts as a capacitor is that for which the NLC impedance remains close to −90°. Figure 6a shows both the modulus and phase of the impedance of *cell 4* as a function of the frequency and for several temperatures. As can be seen, in homeotropic cells, this range is not very large, and it also depends on the temperature. From Figure 6a, it can be seen that this range is between ~200 Hz and 20 kHz depending on the temperature. The study of the capacitive behavior involves another important advantage with respect to a resistance analysis, the linearity. As can be seen in Figure 6b, the resistance decreases with the temperature in the low frequencies range (<1 kHz) while it increases with the temperature (1 kHz< *f* <100 kHz), producing a nonlinear behavior along the frequency range.

**Figure 7:**
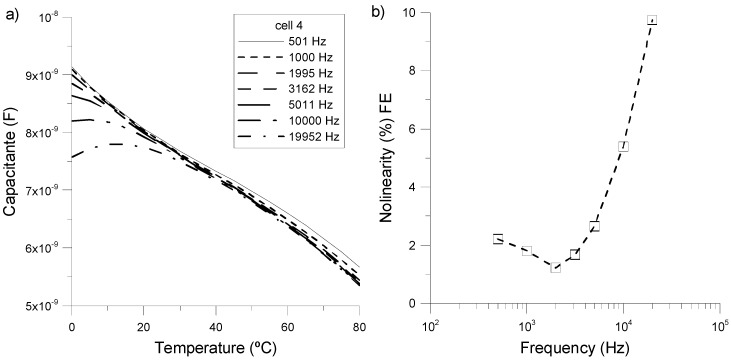
(**a**) Temperature dependence of the capacitance of *cell 4* at various frequencies; (**b**) Non-linearity of *cell 4* as a function of frequency.

Related to the linearity of the measurements, Figure 7 shows the equivalent capacitance of *cell 4* as a function of the temperature for several frequencies of the applied voltage in order to optimize the linearity of our device. The results show a remarkable linearity of the capacitance with the temperature for a large range of frequencies, with the exception of low temperatures (T < 20 °C). In this range, the slope of the curve strongly depends on the frequency. Figure 7b and Table 1 summarize the non-linearity of the results over the full range of temperatures (0 °C–80 °C) as a function of the frequency.

As can be seen, the maximum linearity of the device appears for a frequency of the applied voltage of ~2 kHz, which coincides with the dominant capacitance behavior. In this case, the sensibility is ∆C/∆T = 44.5 pF/°C.

**Table 1:** Non-linearity results presented in Figure 7b.

Frequency (Hz)	Nolinearity (%)
501	2.21
1000	1.8
1995	1.21
3162	1.68
5011	2.65
10,000	5.39
19,952	9.74

### 3.4. Numerical Validation of the Equivalent Electric Circuit

**Figure 8:**
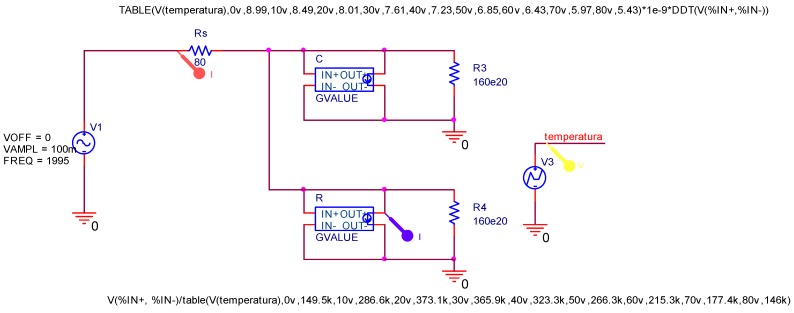
Orcad schematics of the complex equivalent electric circuit of the homeotropic MLC-6290 cell.

Although for an analytical analysis we focused our attention on capacitance measurements, commercial software, such as *ORCAD*, allows the simulation of the complete equivalent electric circuit in order to validate previous results. The simulated device is plotted in Figure 8. The nonlinear capacitor C and resistor R are modeled by using a controlled current source called *GVALUE*. The dependence of the capacitance with the temperature has been specified using a look-up table. This dependent capacitance is then multiplied by the time derivative of the voltage to obtain the output current. On the other hand, the resistance R is nonlinearly mapped from experimental values of Figure 6b. In addition, an independent voltage source *VPWL V3* is the responsible of generating a voltage varying with the temperature; in this case we used a stepped waveform.

The capacitance data obtained from the experimental results were compared with that of the simulated equivalent circuit of homeotropic cells in order to validate the proposed equivalent electric circuit.

**Figure 9:**
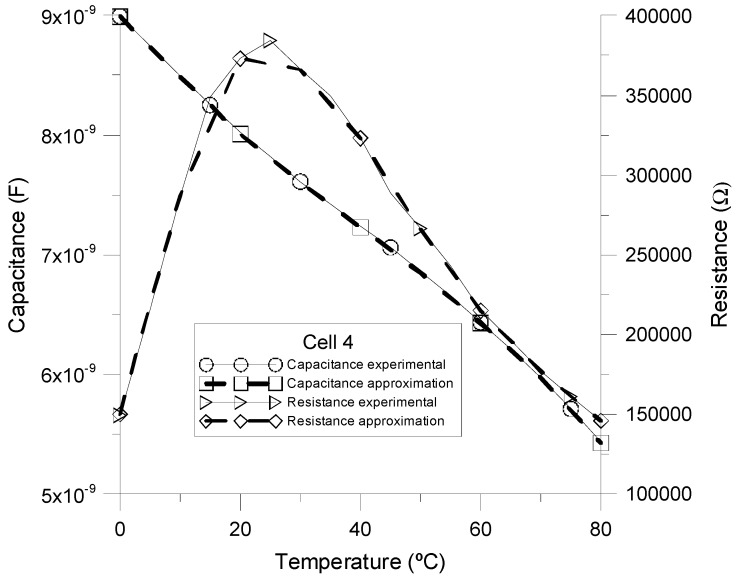
Experimental (solid lines) and simulated (dashed lines) values of the capacitance and resistance of homeotropic MLC-6290 cell.

Figure 9 shows both the experimental and simulated values of the components of the proposed equivalent circuit as a function of temperature for an external frequency of 2 kHz. As can be seen, the results strongly match, concluding that the electric equivalent circuit proposed in Figure 3 satisfactorily represents the behavior of homeotropic NLC cells.

**Figure 10:**
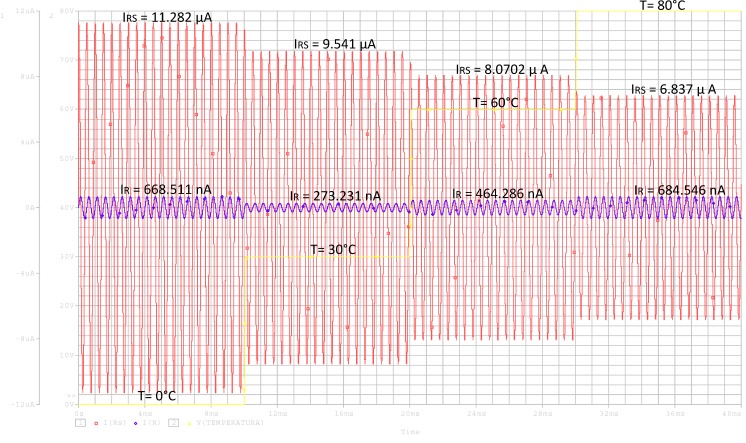
Response of the electrical current through resistance R_S_ (red line) and R (blue line) as a function of the time, while the temperature increases from 0 to 80 °C (yellow line), for the homeotropic MLC-6290 cell.

From the simulations, it is easy to calculate the total sensitivity of the device with the temperature. A sensitivity of ∆I/∆T = 55.56 nA/°C is obtained. Considering that NLC acts as a capacitor (without considering the resistance R), the sensitivity is ∆I/∆T = 55.78 nA/°C. Thus, our previous assumption involves a small deviation of 0.22 nA/°C (0.4%). Figure 10 shows an example of the variation of the electrical current through the circuit as the external temperature changes. The time evolution of currents through R_S_ and R are compared. As can be seen, the current through R is much less than that of R_S_. In conclusion, there is a good agreement between simulation and theory. For further information, we include, in Table 2, the values of the elements of the equivalent circuit for several temperatures and a frequency of 2 kHz.

**Table 2:** Results of fitting in the temperature range of 0 °C to 80°C.

Temperature	Parameter	Value
0 °C	*R* (KΩ)	149.5
	C (nF)	8.99
10 °C	*R* (KΩ)	286.6
	C (nF)	8.49
20 °C	*R* (KΩ)	373.1
	C (nF)	8.01
30 °C	*R* (KΩ)	365.9
	C (nF)	7.61
40 °C	*R* (KΩ)	323.3
	C (nF)	7.23
50 °C	*R* (KΩ)	266.3
	C (nF)	6.85
60°C	*R* (KΩ)	215.3
	C (nF)	6.43
70°C	*R* (KΩ)	177.4
	C (nF)	5.97
80°C	*R* (KΩ)	146
	*C* (nF)	5.43

### 3.5. Proposed Device as Temperature Sensor

Previous results demonstrate the possibility to design a temperature sensor based on the capacitance dependence of a NLC cell. Although various circuits can be used to measure variations of the capacitance, in this section we proposed and implement the device, whose scheme is shown in Figure 10 and that is based on phase measurements. The operating principle of our device is explained below.

Comparing an input controlled sinusoidal voltage (*Es*) and the voltage at the NLC cell, there is a phase shift between the two waveforms that can be expressed as:

(4) $$\Delta \theta \;=\;\arctan\left(2\cdot \pi \cdot f\cdot R_{1}\cdot C_{CL}\right)-90{}^{\circ}$$

The capacitance in the NLC cell has been experimentally measured, as was shown before, and its variation ranged from 5.43 to 8.99 nF (see Table 2). In this case, the maximum sensitivity is achieved using a resistance R_1_ given by:

(5) $$R_{1}=1/(2\cdot \pi \cdot f\cdot \sqrt{C_{CLmin}\cdot C_{CLmax}})$$

Substituting the frequency of the convenient applied voltage (*f =* 1995 Hz) and the extreme values of the NLC capacitance (*C_CLmin_ =* 5.43 nF and *C_CLmax_ =* 8.99 nF) at this frequency, we obtain a resistance of R_1_ = 11.41 MΩ.

The relation between temperature and the phase shift can be described by:

(6) $$\Delta \theta \;=\;-\arctan\left(\left(C_{CLmax}-\;S_{CL}\cdot \left(T-T_{0}\right)\right)/\sqrt{C_{CLmin}\cdot C_{CLmax}}\right)$$

Under our experimental conditions, the phase shift takes values between −37.85° and −52.14°.

**Figure 11:**
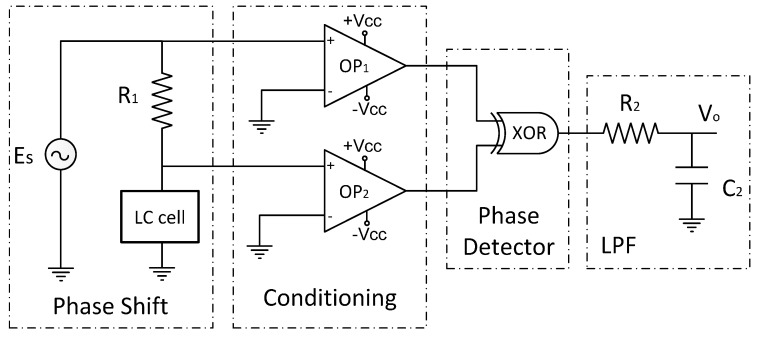
Electronics scheme of a phase-measurement system based on a LC cell.

Many circuits such as PLLs and averaging filter circuits can produce a proportional voltage to the phase shift. In the proposed system (Figure 11), two simple comparators (OP_1_ and OP_2_) produce square waves from the input sinusoidal signals. These digital signals are then compared into an EXOR gate, producing at the output a pulse train (PWM) modulated by the phase shift of the initial signals. A low pass filter (LPF) was also added at the output of the EXOR gate in order to get an average voltage. This voltage can be expressed as:

(7) $$V_{o}=\left(\Delta \;\theta \cdot Vcc\right)/180$$

**Figure 12:**
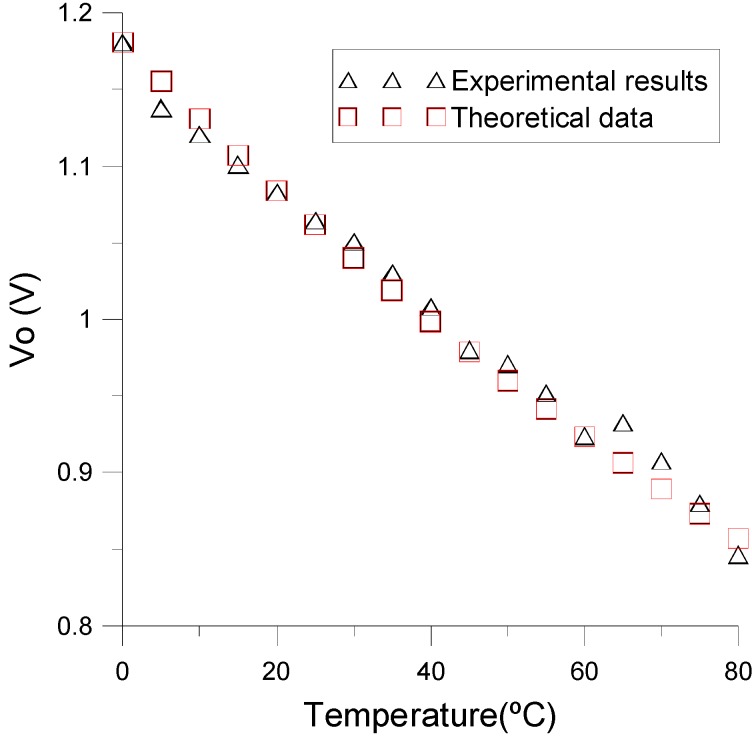
Experimental and theoretical output voltage under different temperatures.

For the design of the low pass filter, we chose a cutoff frequency two decades under the frequency of the pulse train (*f_PWM_)*. This means that the cutoff frequency has to be lower than *f_c_ <* 39 Hz. In addition, the chosen value of the resistor and capacitor was 16 KΩ and 1 μF, respectively. When the temperature is 0 °C, the theoretical value of the output voltage is 1.18 V, which matches with the experimental one. On the other hand, when the temperature is 80 °C, the theoretical output is 0.85 V while the experimental one is 0.84 V. Although they are quite similar, the observed mismatch may be attributed to the component tolerance of the components. The comparison between experimental and theoretical results for intermediate temperatures is shown in Figure 12. As can be seen, the proposed temperature sensor operates linearly (±2%) and accurately (−4.18 mV/°C) in the considered temperature range, following the theoretical considerations. As a summary, Table 3 shows the values of the electronic components used in the phase measurement scheme.

**Table 3:** Values of the electronic components used.

Electronic Component	Value
R_1_	11.41 MΩ
R_2_	16 kΩ
C_2_	1 μF
XOR	7486
OP_1_, OP_2_	UA741

## 4. Conclusions

A novel temperature-phase converter based on the capacitive behavior of liquid crystal cells has been proposed. The temperature dependence of the electrical properties of nematic LC cells has been analyzed, considering either a homeotropic or a planar alignment. An equivalent circuit model has been proposed and experimentally validated. As have been shown from our results, the capacitance of a NLC cell could be accurately used as a temperature-dependent variable. Two important fabrication parameters, the thickness of the cell and the initial alignment, have been studied in order to understand their influence on the temperature sensitivity. Theoretical results show that the sensitivity could be increased by decreasing the thickness, and we have validated it experimentally. In addition, we have shown that homeotropic cells present a larger sensitivity to temperature changes than homogenous ones. We suggest that this important difference could be directly related to the vertical alignment of the LC molecules, even without any external stimulus. Both experimental and simulated results have shown that the proposed device, using a homeotropic LC cell, presents a valid operation in a temperature range from 0 to 80 °C. In addition, we have shown that the sensitivity is not linear within this temperature range. However, our theoretical study demonstrated that it can be improved by selecting an appropriate frequency of the applied voltage. The optimum frequency, which produces a maximum sensitivity, coincides with that at which the NLC cell presents a dominant capacitive behavior along the temperature range. In addition, this involves minimum power consumption, which is an important characteristic for commercial devices. These results were also numerically tested.

Finally, we proposed a new phase measurement scheme to measure the capacitance of a nematic LC cell. A detailed theory to describe the electric behavior of this scheme has been developed and validated through simulations. Interesting effects, like the thermal expansion, the electric heating of the sample or the influence of the spacer in the alignment of the LC, were not considered in this study and they could the motivation of further works.
